# Activation of the pattern recognition receptor NOD1 augments colon cancer metastasis

**DOI:** 10.1007/s13238-019-00687-5

**Published:** 2020-01-19

**Authors:** Henry Y. Jiang, Sara Najmeh, Guy Martel, Elyse MacFadden-Murphy, Raquel Farias, Paul Savage, Arielle Leone, Lucie Roussel, Jonathan Cools-Lartigue, Stephen Gowing, Julie Berube, Betty Giannias, France Bourdeau, Carlos H. F. Chan, Jonathan D. Spicer, Rebecca McClure, Morag Park, Simon Rousseau, Lorenzo E. Ferri

**Affiliations:** 1 Thoracic and Upper GI Cancer Research Laboratories, Research Institute of McGill University Health Centre, 1001 Decarie Boulevard, Block E, Lab #E02-4134, Montreal, QC H4A 3J1 Canada; 2 Department of Experimental Surgery and Department of Surgery, McGill University, 1650 Cedar Avenue, Montreal, QC H3G 1A4 Canada; 3 Meakins-Christie Laboratories, Research Institute of McGill University Health Centre, 1001 Decarie Boulevard, Montreal, QC H4A 3J1 Canada; 4 The Rosalind and Morris Goodman Cancer Research Centre, McGill University, 1160 Pine Avenue, Montreal, QC H3A 1A3 Canada; 5 Department of Surgery, Roy J. and Lucille A. Carver College of Medicine, University of Iowa, University of Iowa Hospitals and Clinics, 200 Hawkins Drive, Iowa City, IA 52242 USA; 6 Department of Pathology, Health Sciences North, 41 Ramsey Lake Road, Sudbury, ON Canada

**Keywords:** NOD1, iE-DAP, ML130, colon cancer, metastasis, cancer-extracellular matrix adhesion, cancer migration, p38 MAPK activation, intravital microscopy, survival analysis

## Abstract

While emerging data suggest nucleotide oligomerization domain receptor 1 (NOD1), a cytoplasmic pattern recognition receptor, may play an important and complementary role in the immune response to bacterial infection, its role in cancer metastasis is entirely unknown. Hence, we sought to determine the effects of NOD1 on metastasis. NOD1 expression in paired human primary colon cancer, human and murine colon cancer cells were determined using immunohistochemistry and immunoblotting (WB). Clinical significance of NOD1 was assessed using TCGA survival data. A series of *in vitro* and *in vivo* functional assays, including adhesion, migration, and metastasis, was conducted to assess the effect of NOD1. C12-iE-DAP, a highly selective NOD1 ligand derived from gram-negative bacteria, was used to activate NOD1. ML130, a specific NOD1 inhibitor, was used to block C12-iE-DAP stimulation. Stable knockdown (KD) of NOD1 in human colon cancer cells (HT29) was constructed with shRNA lentiviral transduction and the functional assays were thus repeated. Lastly, the predominant signaling pathway of NOD1-activation was identified using WB and functional assays in the presence of specific kinase inhibitors. Our data demonstrate that NOD1 is highly expressed in human colorectal cancer (CRC) and human and murine CRC cell lines. Clinically, we demonstrate that this increased NOD1 expression negatively impacts survival in patients with CRC. Subsequently, we identify NOD1 activation by C12-iE-DAP augments CRC cell adhesion, migration and metastasis. These effects are predominantly mediated via the p38 mitogen activated protein kinase (MAPK) pathway. This is the first study implicating NOD1 in cancer metastasis, and thus identifying this receptor as a putative therapeutic target.

**Electronic supplementary material:**

The online version of this article (10.1007/s13238-019-00687-5) contains supplementary material, which is available to authorized users.

## Introduction

Despite advances in chemotherapy and radiation therapy, surgical resection remains the cornerstone of curative treatment for the majority of solid tumors. However, surgery comes at a cost, as post-operative infectious complications are not uncommon. In addition to contributing to immediate post-operative mortality, there is emerging strong clinical and fundamental evidence linking post-operative infection with poor long-term oncologic outcomes for a large number of varied malignancies, including colorectal, lung, and prostate cancers (Law et al., 2007; Hsu et al., 2011; Spicer et al., 2012; Andalib et al., 2013). Indeed, anastomotic leak induced infections after colon cancer surgery doubles the rate of systemic metastases (Law et al., 2007). In order to improve recurrence-free and overall survival in these patients, we and others have previously examined several components of the innate immune system, which is responsible for mediating early inflammatory response secondary to surgery or infection (Schietroma et al., 2013; Sista et al., 2013).

Membranes-bound pattern recognition receptors (PRRs), including toll-like receptor 4 (TLR4) and 2 (TLR2), have been shown in both *in vitro* and *in vivo* studies to enhance metastasis in colon cancers (Hsu et al., 2011; Kim and Karin, 2011; Cario, 2013). However, the inhibition of these receptors do not completely abrogate cancer progression, likely due to the effects of other PRRs which need further investigation in relation to cancer metastasis. Nucleotide-binding oligomerization domain 1 (NOD1) receptor, is a relatively recently described cytosolic PRR known to execute important functions in the immune system (Girardin et al., 2001, Ogura et al., 2001) by recognizing a wide range of microbes bearing the specific meso-diaminopimelic acid (meso-DAP) moiety and mediating the corresponding inflammatory response to these infectious agents (Carneiro et al., 2004). Not only is NOD1 important in host defense and autoimmune diseases, but recently, it has also been implicated in tumorigenesis for a number of cancers, including gastric cancer (Wang et al., 2012), head and neck carcinomas (Millrud et al., 2013), oral squamous cell cancer (Wang et al., 2014), prostate adenocarcinoma (Kang et al., 2012), and lung cancer (Ozbayer et al., 2015).

From these data, it is clear that NOD1 participates in systemic inflammation and infection, whether through its own activation or in conjunction with other PRRs. As well, NOD1 activation has a profound influence in the formation and development of primary tumors in various organs of the body (Kang et al., 2012, Wang et al., 2014). However the role of NOD1 in cancer metastasis is entirely unknown. In the context of resectional surgery for cancer, an understanding of how NOD1 may participate in the inflammation and infection mediated metastasis can ultimately provide new insights for managing malignancies with a curative intent and optimizing clinical outcome.

In this study, we demonstrate high levels of NOD1 receptor expression in primary human CRC adenocarcinoma tissues, as well as multiple CRC cell lines of both human and murine origin to ensure that the results are not the property of one species or one cell line alone. Clinically, we confirm the link between high NOD1 expression and poor overall cancer survival using TCGA mRNA and human tissue microarray (TMA) of CRC. Using a series of *in vivo* and *in vitro* experiments, we determine that the increase in colon cancer adhesion, migration and metastasis is augmented by NOD1 activation via the p38 MAPK pathway. To our knowledge, this is the first study undertaken to implicate NOD1 in infection and inflammation mediated metastasis, its predominant downstream signaling kinase, and its clinical significance in order to identify putative targets to optimize curative surgical therapy.

## RESULTS

### NOD1 is highly expressed in colorectal cancers

**Figure 1:**
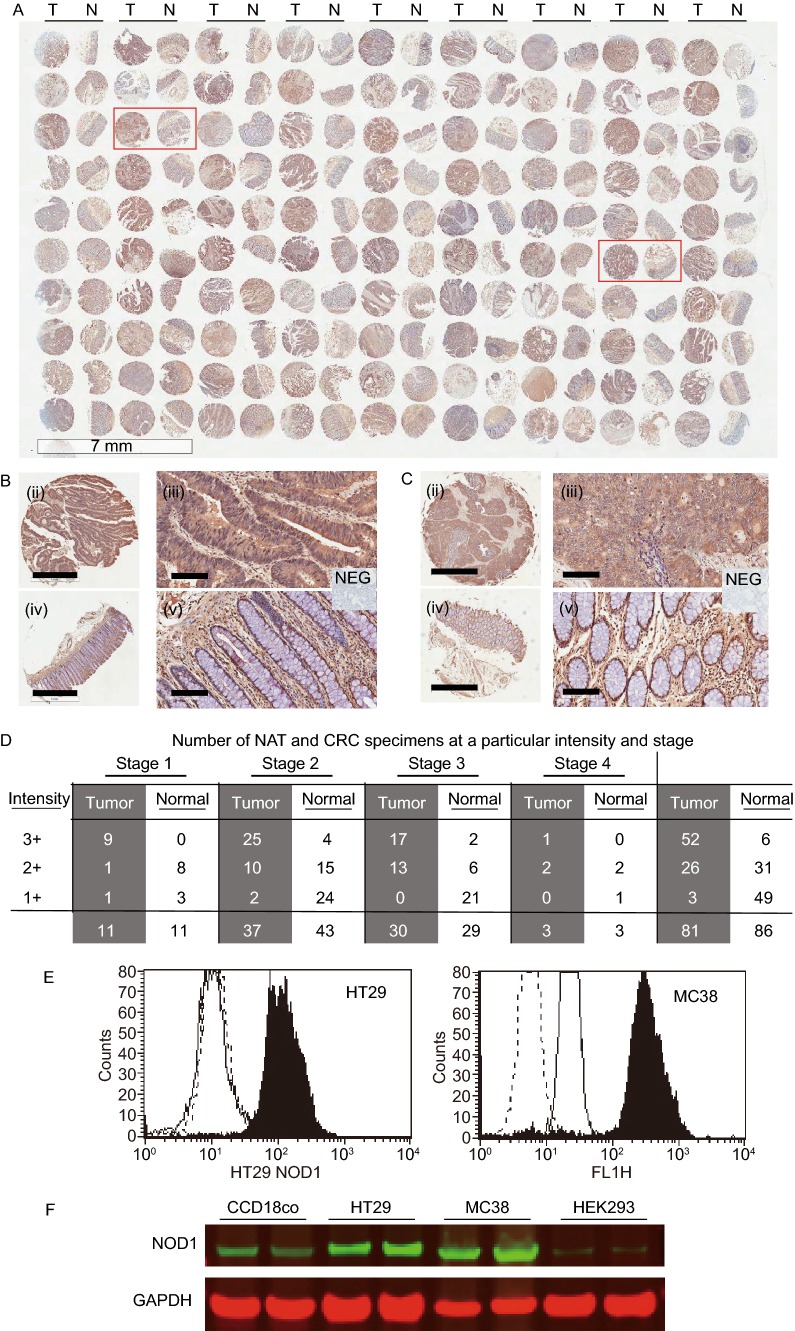
**NOD1 is highly expressed in human colon cancer tissues, cultured cancer cells and liver metastasis**. (A) (i) Immunohistochemical (IHC) staining of human tissue-microarrays (TMA) of early and late stage colorectal adenocarcinoma (CRC) (*n* = 81 for tumor T, and 86 for normal adjacent tissue NAT, scale bar measures 7 μm). (B) Representative images of tumor core (i, ii) and its paired NAT (iii, iv) from one patient are shown at different magnifications. Scale bar measures 1 mm for (i, iii) and 100 μm for (ii, iv). Insert shows negative control (NEG) which Hematoxylin nuclear counterstaining. (C) Representative images of tumor core (i, ii) and its paired NAT (iii, iv) from another patient are shown at different magnifications. Scale bar measures 1 mm for (i, iii) and 100 μm for (ii, iv). Insert shows negative control (NEG). (D) NOD1 intensity in tumor cores and paired NAT by colon cancer stages. Irrespective of stage, there is higher level of NOD1 in primary colon cancer tumors, as evidenced by the greater number of 3+ staining specimens, compare to their NATs (*P* < 0.0001). (E) NOD1 expression in cultured HT29 and MC38 cancer cells is identified using flow cytometry with cytosolic staining technique. NOD1 expression (■), secondary antibody control (—), and unstained cells (- - -) are shown. There is significant shift in peak fluorescence for NOD1 protein in all adenocarcinoma cell lines tested (*P* < 0.05). (F) Immunoblotting is used to confirm the level of NOD1 using whole lysate of the aforementioned cell lines and HEK293 and normal colonic fibroblast tissue CCD18Co, which have low NOD1 expression. The relative NOD1 expression is higher for HT29 and MC38 than HEK293 and CCD18Co

NOD1 expression has been consistently demonstrated in various normal human tissues including nasopharynx, breast, lungs, intestines and prostate, and its expression profile in cancerous tissues can be highly variable (Kang et al., 2012; Millrud et al., 2013; Wang et al., 2014). In order to confirm the presence of NOD1 in primary human CRC, immunohistochemistry (IHC) was carried out on tissue microarrays (TMA) of colorectal adenocarcinomas of both early and advanced stages probing for NOD1. Cytosolic NOD1 staining was graded by intensity of staining ranging from 1+ to 3+ by an independent pathologist. Irrespective of CRC stages, tumor cores were associated with increased NOD1 protein levels compared to that of paired normal adjacent tissues (Cochran-Mantel-Haenszel *P* < 0.0001) (Fig. 1A–C). Out of 81 cases of CRC on the TMA, 52 cores (64%) had 3+ NOD1 intensity, 26 cores (32%) had 2+ intensity and 3 cores (4%) had 1+ or no staining. In the paired normal adjacent tissues (NAT), the trend was reversed as 6 out of 86 cores (7%) had 3+ NOD1 intensity, 31 cores (36%) had 2+ intensity and 49 cores (57%) had 1+ or no staining. This trend was observed at all stages of CRC (Fig. 1D).

In addition to its positive expression in human tumors, NOD1 was also highly expressed in cultured CRC cell lines. Both human (HT29) and murine (MC38) CRC cells were tested to ensure that the expression pattern was not the property of a single cell line or species. Using flow cytometry with intracellular staining technique, we demonstrated a consistent expression of NOD1 receptor in both human and murine CRC cells (Fig. 1E). The existence of NOD1 receptors in cancer cells was further validated using conventional immunoblotting technique. HT29 and MC38 had higher levels of NOD1 compared to non-cancerous human colonic fibroblasts CCD18co and immortalized embryonic HEK293 cell line, in which NOD1 expression was low (Fig. 1F).

### Clinical impact of NOD1 on survival

Although NOD1 expression is elevated in colon tumors and liver metastases, whether these elevated expressions exert any clinical significance in patients with CRC remains unproven. Hence, we examined the effects of increased NOD1 mRNA and protein levels on survival. This was achieved using TCGA RNA-Seq data and human TMA immunohistochemistry of CRC adenocarcinomas. Clinical data from both TCGA and human TMA were available for correlating NOD1 with overall survival (OS).

**Figure 2:**
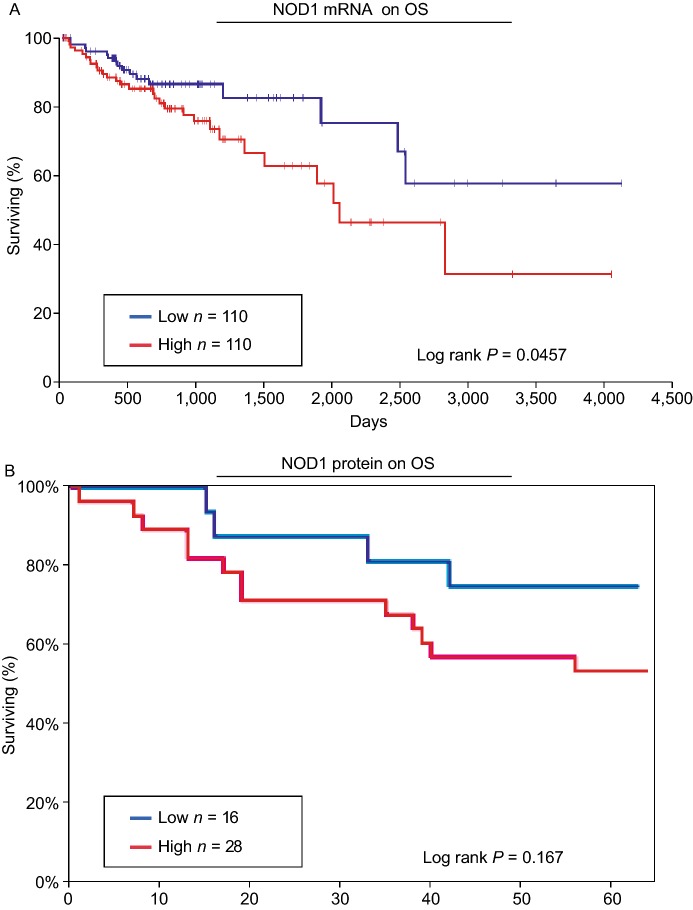
**High NOD1 expression is associated with decreased overall survival in patients with colorectal cancers**. (A) Using OncoLnc, TCGA RNA-Seq COAD data is analyzed by correlating overall survival with NOD1 expression levels (High vs. Low). Having high levels of NOD1 is associated with poorer survival (Log rank *P* = 0.0457). The “High” and “Low” NOD1 expression represents non-overlapping top and bottom 25% NOD1-expressing tumors, respectively. (B) Using TMA of human CRC, overall survival data is analyzed by correlating them with changes in NOD1 protein expression levels (High vs. Low). There is no significant association between high levels of NOD1 and overall survival (*P* = 0.167) based on our current data. The “High” and “Low” NOD1 expression represents non-overlapping tumor cores with 2+ and 0+ change in NOD1 intensity compared to baseline, respectively

Using OncoLnc®, an analysis tool designed to assess TCGA clinical data (Anaya, 2016), we determined the significance of NOD1 mRNA expression on OS in patients with CRC. Non-overlapping top and bottom 25% (*n* = 110 in each comparison group) of the TCGA CRC adenocarcinoma (COAD) dataset were used for the Kaplan-Meier comparison. We found a significant reduction in CRC OS with increasing NOD1 mRNA levels (*P* = 0.0457) (Fig. 2A). Using data from the whole TCGA COAD dataset (*n* = 440), the Cox regression coefficient for NOD1 on CRC was 0.26 (*P* = 0.0120).

For NOD1 IHC study, OS data corresponding to human CRC TMA previously shown in Fig. 1A, was used to determine NOD1 expression on colorectal cancer patient survival. For this analysis, change in NOD1 intensity was used. This adjustment was applied to remove any baseline effects of NOD1 CRC compared to paired NAT. Kaplan-Meier survival analysis was applied on the two groups, low (*n* =28) vs. high (*n* =16) NOD1 expression (Fig. 2B). Our results did not revealed any significant relationship with OS (*P* = 0.167), likely related to relatively low sample size. However, we observe a trend of lower survival rate with higher levels of NOD1.

### NOD1 activation augments *in vivo* colon cancer metastases

**Figure 3:**
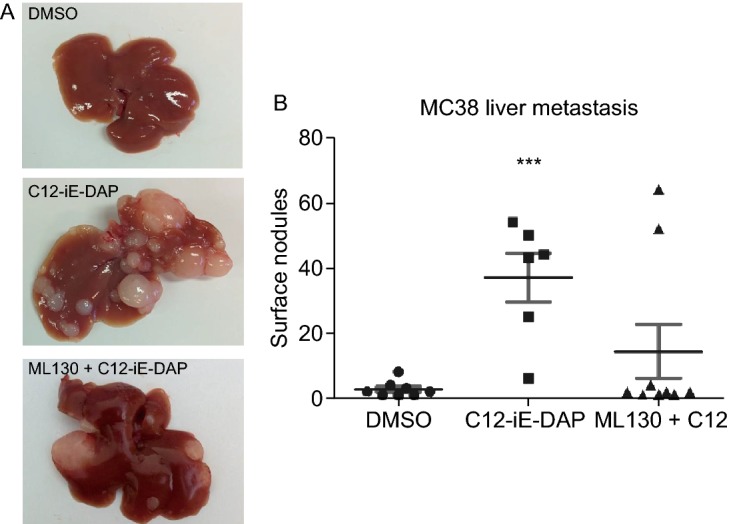
**NOD1 stimulation provokes*in vivo*hepatic metastasis of colon cancer cells**. (A) Representative murine liver samples with metastasis for each condition are shown. Compared to DMSO control (left), C12-iE-DAP (2,000 ng/mL) (middle) stimulation of MC38 colon cancer augments their metastasis. This augmentation is abrogated with ML130 (10 μmol/L) co-incubation (right). (B) Results from 2 independent experiments (*n* = 7 mice/condition) are quantified by counting the number of surface liver nodules post-necropsy. Only 8/14 mice in the DMSO group, 6/14 mice in the C12 group, and 9/14 mice in the ML130 + C12 group survived until 21 days post intrasplenic cancer cell injection for inclusion in the figure. Mice that died prior to the endo-point did not have any liver metastasis on necrosectomy. M and SEM are shown. All comparisons are made with respect to the DMSO control. Only significant comparisons are labelled. “***” denotes *P* < 0.0001

Increased expression does not necessitate increased functional activities of the NOD1 receptors. Hence, after confirming the clinical significance of NOD1 over-expression in colon cancer, direct evidence on whether NOD1 activation augments metastasis was investigated. C12-iE-DAP (henceforth known as C12), a highly specific NOD1 agonist derived from gram-negative bacterial cell wall, and ML130, the specific small chemical antagonist of NOD1, were used to activate and inhibit NOD1, respectively. The *in vivo* effects of NOD1 activation with C12 and co-incubation with ML130 were assessed by injecting thus treated murine MC38 colon cancer cells intrasplenically into wildtype C57BL6 mice. DMSO was used as the vehicle control. After 21 days, necropsy was performed and gross metastasis was quantified by counting the total hepatic surface nodules. We observed a significantly higher number of CRC metastatic deposits in the C12 stimulated group (37 ± 7) compared to DMSO control (3 ± 1) and ML130 co-incubated group (14 ± 8) (*P* = 0.0279) (Fig. 3). NOD1 activation indeed augments CRC metastasis, and this effect can be attenuated by NOD1 inhibition.

### NOD1 activation increases ECM adhesion and cancer migration

**Figure 4:**
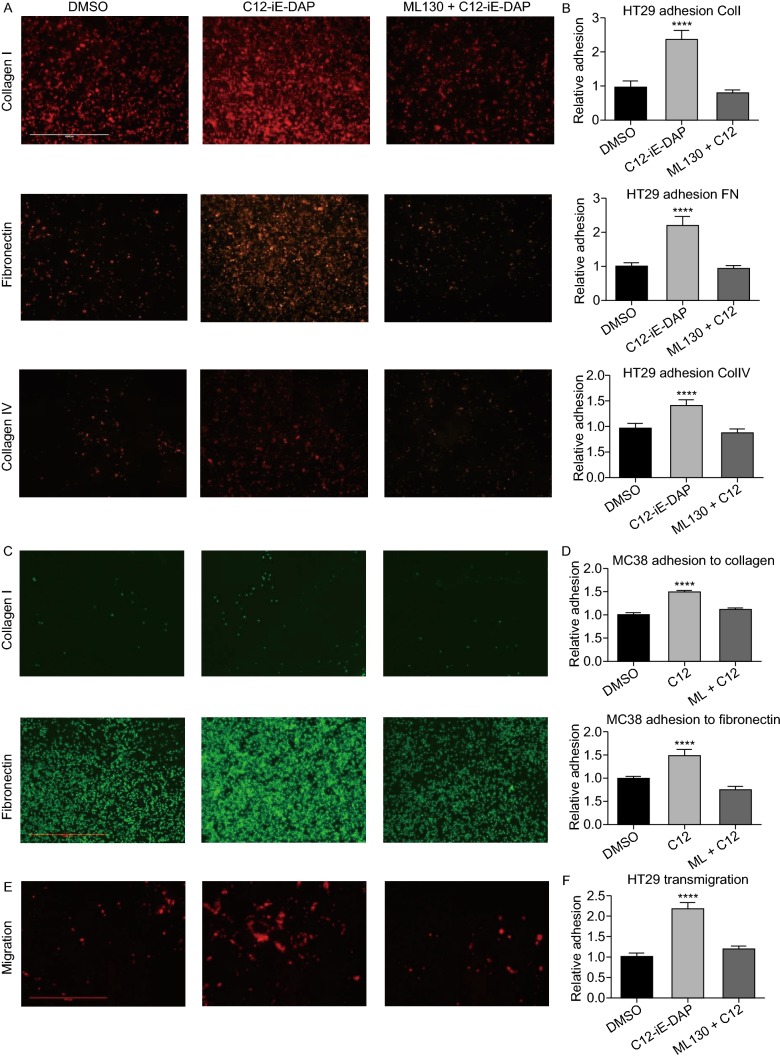
**C12-iE-DAP induced NOD1 activation also enhances*in vitro*adhesion and migration of colon cancer cells**. (A) Representative images of HT29 adhering to collagen I, IV and fibronectin. Bright intensities represent RFP-expressing HT29 cells. There is a marked increase in adhesion upon C12-iE-DAP stimulation compared to DMSO control and ML130 co-incubation. (B) Quantification of adhesion using crystal-violent-colorimetric method shows significant increase in HT29 adhesion to fibronectin (2-fold), collagen I (2-fold) and IV (1.5-fold) compared to baseline. These increases are abolished with the co-incubation of ML130. (C) MC38 adhesion to collagen I and fibronectin under DMSO, C12 and ML130 + C12 treatments are done to ensure that the effect of NOD1 on HT29 is not specific to cell line of one species. (D) There is a significant increase in MC38 adhesion to collagen I (1.5-fold) and fibronectin (1.5-fold) compared to baseline. These increases are again abolished with the co-incubation of ML130. (E) Similarly, C12-mediated NOD1 activation also increases simple migration of RFP-expressing HT29 cells in a Boyden chamber model. (F) Quantification of migration across the Boyden membrane shows a 2-fold increase in NOD1 activated cancer cells compared to DMSO and ML130 co-incubation (*P* < 0.0001). (G) Scale bar measures 1mm and applies to all microscopy images in Fig. 4. Error bars represent SEM. A minimum of 3 repeats each with 8 replicates are done for each ECM and 3 replicates for migration assays. All comparisons are made with respect to the DMSO control. Only significant comparisons are labelled. “***” denotes *P* < 0.0001

After identifying the involvement of NOD1 in mediating metastasis, we then examined the steps necessary to achieve this augmentation. There is ample evidence to suggest that tumor cells can attach to various components of the ECM during metastatic dissemination (Ryschich et al., 2009; Campbell et al., 2010). Hence, we surmised that C12 mediated NOD1 activation must be involved in circulating tumor cell (CTC) interaction with the ECM, especially, collagens and fibronectins, which are found in abundance in various organs and structures, including the subendothelial layers of blood vessels and basement membranes of various tissues (Kramer et al., 1980; Liu et al., 1990; Whelan and Senger, 2003). Indeed, with NOD1 activation, we observed a significant increase in *in vitro* static adhesion of human colon cancer cells HT29 to fibronectin (2-fold), collagen I (2-fold) and collagen IV (1.5-fold) compared to baseline DMSO control (*P* < 0.0001) (Fig. 4A and 4B). These pro-adhesive phenotypes were completely attenuated in the presence of ML130, a specific NOD1 inhibitor. This pattern was also observed with murine MC38 cancer cells (Fig. 4C and 4D), suggesting a generalized mechanism in adhesion upon NOD1 activation. We examined the effect of NOD1 activation on migration using HT29 in a Boyden chamber model, and similarly observed an increase in migration with C12 induced NOD1 activation (Fig. 4E and 4F). Again, this increase in colon cancer migration was abrogated with ML130 co-incubation.

### NOD1 mediates *in vivo* colon cancer adhesion to liver sinusoids

**Figure 5:**
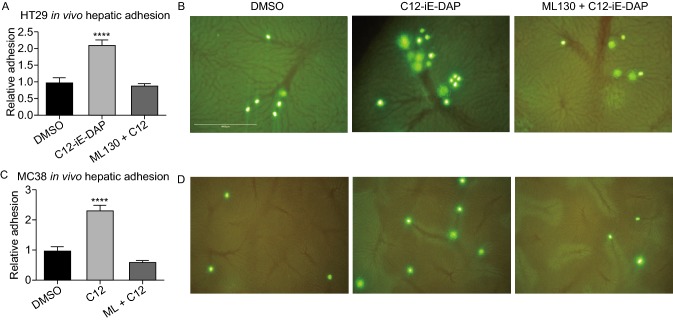
**NOD1 stimulation increases*in vivo*hepatic sinusoid adhesion of circulating tumor cells (CTCs)**. (A) Relative *in vivo* adhesion of HT29 sinusoids under DMSO, C12 stimulation (2,000 ng/mL), and ML130 co-incubation (10 μmol/L) (*n* = 8 mice/condition). NOD1 activation leads to a 2-fold increase in *in vivo* hepatic adhesion compared to DMSO or ML130 co-incubation (*P* < 0.0001). (B) Representative microscopy images of HT29 adhesion to hepatic sinusoids under DMSO, C12, and ML130 + C12 are shown. Green intensities represent HT29 cells live-stained with CSFE and the reticular patterns represent hepatic sinusoids. Compared to DMSO (left), C12-induced (middle) NOD1 activation leads to enhanced hepatic sinusoid adhesion. Such enhanced adhesion is absent when co-incubated with ML130 (right). (C) To show a generalized effect of NOD1 activation on hepatic adhesion, the above experiment is repeated using murine colon cancer MC38 (*n* = 5 mice/condition). The same results are obtained in which C12 stimulated cells (middle) showed 2-fold increase in *in vivo* adhesion. (D) Representative images of MC38 adhesion to hepatic sinusoids are shown. (E) Scale bar measures 1mm and applies to all microscopy images in Fig. 5. Error bars represent SEM. All comparisons are made with respect to the DMSO control. Only significant comparisons are labelled. “***” denotes *P* < 0.0001

Although we clearly demonstrated that NOD1 activation increases the migratory phenotype of colon cancer cells *in vitro*, we sought to confirm its role in promoting a metastatic phenotype in a more physiologically relevant *in vivo* model. The *in vivo* adhesive properties of NOD1 activation in CRCs were examined using hepatic intravital microscopy (IVM). After injecting NOD1 stimulated human HT29 or murine MC38 cancer cells into the spleen of wildtype C57BL6 mice, the *in vivo* adhesion to hepatic sinusoids was documented in real time as we have previously performed (Hsu, et al., 2011). With NOD1 activation, a 2-fold increase (*P* < 0.0001) in *in vivo* adhesion of both HT29 (Fig. 5A and 5B) and MC38 (Fig. 5C and 5D) to hepatic sinusoids was observed. This response was completely abrogated with the addition of specific NOD1 inhibitor, ML130 (Fig. 5A and 5C). These data suggest that NOD1 activation is important for *in vivo* CRC adhesion, another key step in metastatic progression.

### Knockdown validation of the role of NOD1 in cancer metastasis

**Figure 6:**
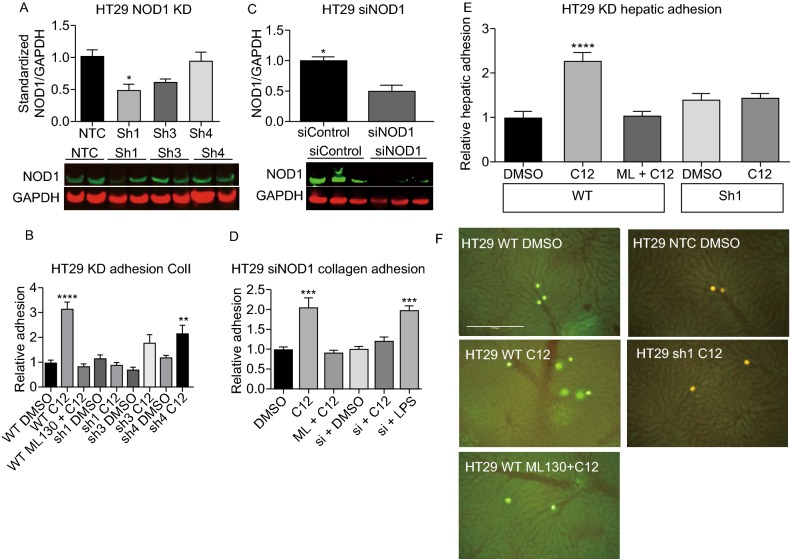
**NOD1 knockdown (KD) cell lines abrogate the pro-metastatic effects of NOD1 stimulation by C12-iE-DAP**. (A) Subcultures of HT29 are created using shRNA with puromycin selection. On immunoblotting, the level of NOD1 remaining in Sh1, Sh3 and Sh4 are 48% ± 10%, 60% ± 6% and 94% ± 14%, respectively compared to 100% ± 12% for the non-targeting control (NTC) (*P* = 0.0255). (B) Wildtype and knockdown HT29 cells lines are subjected to a static *in vitro* collagen I adhesion assay to determine their phenotype when stimulated with C12. Sh1 fails to respond to C12 stimulation and demonstrates low adhesion comparable to control (*P* < 0.0001). In contrast, Sh4, which has the same level of NOD1 as NTC, is still able to mediate full C12 response. (C) To ensure that the observed knockdown of NOD1 in Sh1 is not due to non-specific effects of lentiviral particles, we also use siRNA to transiently reduce NOD1 expression, and obtain similar results of NOD1 reduction (49% ± 9% remaining) compared to siControl (*P* = 0.0443). (D) siRNA knockdown adhesion to collagen I is subsequently performed. The results are similar to those of Sh1 adhesion with siNOD1 lacking a response to C12 stimulation (*P* < 0.0001). (E) Furthermore, through *in vivo* hepatic adhesion assay (*n* = 5 mice/condition) showed a 2-fold increase in for wildtype + C12, but not for DMSO or NTC controls or Sh1 cell lines (*P* < 0.0001). (E) Representative images are shown for *in vivo* hepatic adhesion assay. Bright intensities represent either CSFE labeled (wildtype HT29) or RFP tagged (NTC and KD HT29) cells. Scale bar measures 1 mm. Error bars represent SEM. All comparisons are made with respect to the DMSO, siControl, or NTC control. Only significant comparisons are labelled. “***” denotes *P* < 0.0001, “****” denotes *P* < 0.00001

In order to more specifically delineate the effects of NOD1 activation on the metastatic phenotype, knockdown cells were created. Lentiviral transduction was used to create stable shRNA knockdown cell lines on a HT29 wildtype background. Of three knockdown constructs (sh1-NOD1, sh3-NOD1, sh4-NOD1; henceforth known as Sh1, Sh3 and Sh4) tested, only Sh1 produced a significant reduction in NOD1 protein level compared to non-targeting control (NTC) (Fig. 6A). The amount of protein remaining in Sh1, Sh3 and Sh4 were 48% ± 10%, 60% ± 6% and 94% ± 14% compared to non-treated control (NTC), 100% ± 12%, on immunoblotting (Fig. 6A). We then validated the phenotype of knockdown cell lines through functional assays. Compared to wildtype (WT), Sh1 failed to mount a response to C12-induced NOD1 activation and showed consistently lower adhesion to collagen I compared to Sh1 + DMSO and wildtype + DMSO controls (Fig. 6B). C12 stimulation of Sh4-HT29 cancer cells, which was used as a negative control as this construct elicited the same NOD1 protein expression as non-treated controls, a phenotype as adhesive as wildtype + C12 was observed (Fig. 6B). These data confirm our prior results and imply that NOD1 levels are important for mediating C12 induced cancer cell adhesion *in vitro*.

To ensure that the results from the Sh1 were not secondary to off-target effects of the lentivirus, a second method of knockdown was used. HT29 wildtype cells were incubated with siRNA against NOD1 mRNA and its expression level was successfully down-regulated as determined by immunoblot analysis (Fig. 6C). Similar to the above knockdown experiments, the reduced level of NOD1 protein completely abolished the C12-induced, but not LPS-induced, adhesiveness of HT29 cells to collagen I (Fig. 6D).

Because of its stability of knockdown, we selected the lentiviral-transduced Sh1 NOD1-knockdown cell line for subsequent *in vivo* experiment. Murine hepatic adhesion assay revealed that Sh1 was less adhesive compared to wildtype or NTC in the presence of C12 (Fig. 6E and 6F). Taken together, these *in vitro* and *in vivo* knockdown data suggest that the effects of C12 activation and ML130 inhibition are indeed mediated through NOD1, and that NOD1 serves an important function in CRC metastasis.

### NOD1-augmented colon cancer adhesive phenotype is p38 MAPK dependent

**Figure 7:**
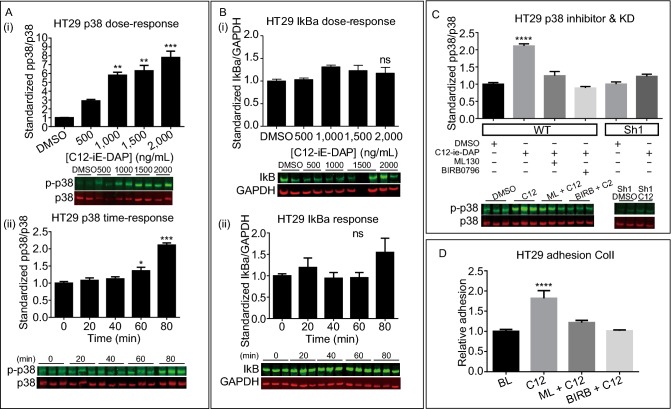
**The metastatic effects of NOD1 activation is mediated through p38 MAPK**. (A) C12-induced NOD1 activation leads to both a dose- and time-dependent p38 activation (*P* = 0.0007 and < 0.0001, respectively) of up to 7-fold from baseline. (B) Such a response is not observed with NF-κB activation via IkBa degradation (*P* = 0.2311, 0.6810 respectively). (C) Phosphorylation of p38 is at baseline with ML130, BIRB0796 co-incubation (*P* < 0.0001). HT29sh1 knockdown cells also show no significant p38 phosphorylation upon C12 stimulation (*P* < 0.0001). (D) Static *in vitro* adhesion assay to collagen I was used to verify the effect of p38 on adhesion using HT29. Decrease in adhesion is observed in group with BIRB0696 co-incubation, further conferring a downstream role of p38 in NOD1 metastasis pathway (*P* < 0.0001). At least 2 independent experiments, each with *n* = 2 replicates/condition, are conducted per signaling figure. For BIRB0796 adhesion, 2 experiments each with *n* = 4 are performed. M ± SEM is reported. All comparisons are made with respect to the DMSO control. Significant comparisons are labelled. “*” denotes *P* < 0.05, “**” denotes *P* < 0.001, “***” denotes *P* < 0.0001. “ns” represents no significance

Although the pattern of NOD1 signaling in normal cells is well established, very little is known of its downstream signaling in cancer cells (Benko et al., 2008; Berube et al., 2009; Lappas, 2013; Zhang et al., 2014). We thus sought out to determine the major signaling pathway in CRC upon NOD1 activation. It has been shown that inflammatory response from NOD1 activation is mediated by nuclear translocation of NF-κB p65 subunit and/or phosphorylation of p38 (Berube et al., 2009; del Barco Barrantes and Nebreda, 2012). Hence, we started by focusing on these two signaling pathways in CRC. We observed a significant, up to a 7-fold increase in p38 phosphorylation with increasing doses of C12-iE-DAP up to 2,000 ng/mL (*P* = 0.0007) and with increasing incubation time up to 80 min (*P* < 0.0001) (Fig. 7A-i and ii). Such dose and time dependent activation (*P* = 0.6810, 0.2311 respectively) were not seen with respect to NF-κB as seen indirectly via IkBa degradation (Fig. 7B-i and ii). Hence, we surmised that p38 MAPK was more important for NOD1 downstream signaling in CRC cancer cells. Accordingly, we examined the amount of p38 phosphorylation in the presence of ML130 and its specific kinase inhibitor BIRB0796, as well as using Sh1 HT29 knockdown cell line. The p38 phosphorylation in HT29 treated with C12 were significantly higher compared to HT29 DMSO control, HT29 co-incubated with ML130 or BIRB0796, or Sh-NOD1 knockdown treated with C12 (*P* < 0.0001) (Fig. 7C). Similar results were identified using murine MC38 colon cancer cell line to ensure that the observed effects on p38 MAPK were not the property of a single cell line or species (Fig. S1). Finally, to ensure that the observed biochemical changes in p38 correlate with phenotypes, we performed an *in vitro* collagen I adhesion assay using HT29 cells treated with C12 and the p38 inhibitor BIRB0796. Only basal adhesion level was seen in the group with BIRB0796 co-incubation (Fig. 7D). Based on these data, we conclude that downstream signaling and adhesive properties of NOD1 activation is p38 dependent in HT29 cancer cells.

## Discussion

Mounting evidence demonstrates an association between post-operative infections and cancer recurrence (Hsu et al., 2011; Spicer et al., 2012; Andalib et al., 2013; Cools-Lartigue et al., 2013). Our group has previously investigated several possible mechanisms to explain this infection-facilitated cancer metastasis paradigm, including the role of immune cells (Spicer et al., 2012; Cools-Lartigue et al., 2013). However, pattern recognition receptors (PRRs) represent another attractive target to explore this phenomenon further as they are the first host molecules to interact with invading infectious pathogens. NOD1, a relatively new PRR that recognizes iE-DAP derived from gram-negative and gram-positive bacterial wall, plays an important role in host defense and inflammation and has recently been shown to promote tumor development (Girardin et al., 2001). However, its role in metastasis and its potential as a therapeutic target are entirely unknown. In this study, we have, for the first time, established a direct link between NOD1 activation and metastasis of colon cancer (Haggar and Boushey, 2009).

NOD1 is widely expressed in normal human tissues, including the epithelium of intestines, lungs and prostate (Kang et al., 2012; Wang et al., 2012). It is thus not surprising to see that malignancies that originate from these epithelial cells may retain NOD1 expression, as we have demonstrated with IHC analysis of colon cancer tumors in patients undergoing resection. In addition, multiple colon cancer cell lines of both human and murine origin, including HT29 and MC38, are also shown to express abundant amounts of NOD1.

Despite its ubiquitous expression in colonic tumors and liver metastases, the clinical impact of NOD1 need to be established. We use NOD1 mRNA and protein levels obtained from RNA-Seq in TCGA and intensity from IHC analysis of TMA containing human CRC tissue. TCGA COAD data and human CRC tumor IHC demonstrate a significant survival trend between high NOD1 levels and lower overall survival. Ideally, we would have correlated NOD1 levels to disease recurrence which more accurately reflects metastasis, however disease-free survival and recurrence data are not captured by TCGA or for the patients within the TMA that we had at our disposal.

Elevated NOD1 expression does not oblige receptor activation. Having determined NOD1 expression and its clinical impact on colon cancer survival at the clinical level, its mechanistic role in metastasis is investigated through its activation and inhibition in a series of functional assays.

The *in vivo* metastasis model employed in this manuscript replicates an intraoperative phenomenon in which CRC cancer cells disseminate to the liver via portal vein during colon resection (Weitz et al., 2000; Koch et al., 2001; Papavasiliou et al., 2010). As CRC often metastasize to the liver via the portal circulation, intrasplenic injection of cancer cells closely mimics this process and allows for the examination of the CRC metastasis under NOD1 activation and inhibition. Mice injected with C12-treated MC38 cells develop more metastatic nodules in their liver compared to DMSO vehicle control-treated cells, an effect that is also eliminated with ML130. We choose to study *in vivo* metastasis using only murine MC38 cell line as the use of human HT29 cell line would require athymic nude or SCID mice. The altered immune profile of these mice would confound our study on inflammation-mediated metastasis (Richmond and Su, 2008). However, for *in vitro* or transient *in vivo* study, we make use of the human HT29 cell line as it is more relevant to the human disease process.

Having identified a role for C12 in mediating metastasis, we needed to determine the necessary processes culminating to the observed metastatic phenotype. It is well accepted that adhesion to ECM is a crucial step in cancer progression (Ryschich et al., 2009; Campbell et al., 2010). We and others have previously shown that bacterial endotoxins, such as LPS, can alter the adhesive abilities of cancer cells towards various components of the ECM (Hsu et al., 2011); hence, we seek to determine the importance of C12 in *in vitro* adhesion of CRC cancer cells. Collagen I is selected as one of the ECM substrates for its abundance and wide distribution in human tissues, and fibronectin and collagen IV are selected for their abundance in the basement membranes which are important for tumor invasion and extravasation from bloodstream into tissues (Hou et al., 2011). Likewise, migration is another important step in metastasis, which depends on the ability of CTCs to move through bloodstream and tissues. Hence, NOD1 activation enables cancer cells to fulfill two key steps in metastasis: adhesion and migration.

Furthermore, using intravital microscopy (IVM), which allows for visualization of living cancer cells migrating through blood vessels and adhering to them in a more physiologically relevant model of the early steps of hematogenous metastasis, we determine that NOD1 activation can increase adhesion of circulating HT29 and MC38 cells to hepatic sinusoids, an effect that is eliminated with ML130 addition. These *in vivo* data strengthen results from the aforementioned experiments, in which enhanced adhesion is thought to be an important mechanism for NOD1-induced metastasis.

Knockdown is used as a method of studying the effects of proteins through their depletions. While lentiviral shRNA provides a stable means for achieving knockdown, not every construct is effective and some nonspecific gene disruption by knockdown can be lethal to cells (Matsui et al., 2006; Feng et al., 2010). Of the 3 lentiviral shRNA constructs tested, Sh3 and Sh4 do not demonstrate a decrease in cell adhesion to *in vitro* ECM or *in vivo* endothelia. Sh1 achieves successful knockdown and is verified to have decreased NOD1 protein levels. To further ensure that the observed knockdown is not due to non-specific effects of the lentiviral particle or its components, we also examine NOD1 knockdown using siRNA and obtain similar results. Additionally, the shRNA and siRNA knockdown cell lines both lack response to C12 stimulation in *in vitro* or *in vivo* adhesion assays to ECM or endothelia. These suggest NOD1 is indeed important in mediating C12 response.

In normal epithelial cells, inflammatory response of NOD1 to microbial stimuli is mediated by the NF-κB and/or p38 MAPK pathways (Kobayashi et al., 2002; Correa et al., 2012). In the CRC cell lines examined in our study, p38 MAPK is identified as the predominant signaling cascade upon C12-induced NOD1 activation. We observe both a time- and C12 dose-dependent phosphorylation of p38. These increases in p38 phosphorylation are attenuated by the addition of ML130 and BIRB0796. Such biochemical changes in p38 can also be correlated at the phenotype level, in which an increase in adhesion of HT29 and MC38 cells to collagen I occurs with C12 incubation, dissipates with ML130 and BIRB0796 co-incubation. Corroborating data from the literature have long documented that p38 activation can be induced by endotoxins (Han et al., 1993) and there is ample evidence to implicate p38 in metastasis of several cancers, including lung, gastric and prostate cancers (del Barco Barrantes and Nebreda, 2012). Consequences of p38 activation include upregulation of adhesion molecules, modulation of focal adhesion complexes via pp38-vinculin interaction, release of inflammatory cytokines (del Barco Barrantes and Nebreda, 2012). These can increase CTC adhesion, migration and invasion into target organs as we have observed from our *in vitro* and *in vivo* experiments. In addition, p38 is also implicated in the survival and proliferation of micrometastasis (Wada and Penninger, 2004). However, we are not able to demonstrate any difference in cell proliferation among baseline, C12 and ML130 + C12, nor are we able to show increase in adhesion-free survival with these treatment groups (Figs. S2 and S3), suggesting NOD1-p38 axis must employ an alternative mechanism in mediating metastasis, which remains to be elucidated.

This research explores the role of NOD1 receptor, a member of the innate immune PRR, in cancer metastasis, an entirely novel paradigm. We have demonstrated NOD1 as novel targets in inflammation-mediated cancer metastasis. In this study, we are able to attenuate the effects of C12-induced NOD1 activation in an infection/inflammation model of cancer metastasis at two different levels: NOD1 at the receptor level with ML130 and p38 the major signaling molecule with BIRB0796. Coupled with survival analyses from RNA-Seq and TMA immunohistochemistry, these data strongly argue a role for NOD1 in infection-mediated cancer metastasis and thereby clinical recurrence. Further studies of the NOD1 downstream effectors is needed in order to identify additional putative therapeutic targets to reduce recurrence in patients undergoing resection with curative intent.

## MATERIALS AND METHODS

### Reagents

NOD1 agonist (C12-gamma-D-glutamyl-meso-diaminopimelic acid, or C12-iE-DAP) and specific inhibitors to NOD1 (ML130), p38 (BIRB0796), ERK1/2 (PD184352), NF-κB (Bay117082) were purchased from Invivogen and Tocris-Bioscience. Collagen I (rat), collagen IV (human), and fibronectin (human), carboxyfluorescein-succinimidyl ester (CSFE) and DMSO was purchased from Roche, Invivogen and Sigma.

### Cell culture

Human and murine CRC cell lines (HT29, MC38) and normal human colonic fibroblast (CCD18co) were cultured in AMEM media (Wisent) supplemented with 10% fetal bovine serum (FBS) and 1% penicillin/streptomycin (PS). HEK293 cells were cultured in DMEM (Wisent) with 10% FBS and 1% PS. LHC basal medium (Gibco) with complete mineral supplements and 1% PS were used for serum starvation of these cells. All cells were incubated at 37 °C with 5% CO_2_.

### Stimulation and inhibition

NOD1, p38, and NF-κB were inhibited with ML130 (2–10 μmol/L), and BIRB0796 (0.2–0.8 μmol/L), respectively. Thereafter, C12-iE-DAP was added (20–4,000 ng/mL) and incubated over various time intervals (15 min–24 h) to obtain optimized functional phenotype and signaling parameters. DMSO and sterile water were used as vehicle controls. For subsequent adhesion, intravital microscopy (IVM) and metastasis experiments, the optimal treatment time used was 30 min for ML130 or DMSO control followed by incubation with C12-iE-DAP or sterile water control for another 60 min.

### Antibodies

Primary antibodies used were: NOD1 (Bioss#bs-7085R), p38 (CST#9228S), pp38 (Millipore#09-272), IkBa (Upstate-Biotechnology#06-494), and GAPDH (Millipore#MAB374). Secondary antibodies for flow cytometry and immunoblotting were: DyLight800-anti-rabbit-IgG (ThermoScientific#35571), DyLight680-anti-mouse-IgG (ThermoScientific#35518), Alexa Fluor A647 (MolecularProbes#A21236), FITC-conjugated anti-mouse (Cedarlane#CLCC30001) and anti-rabbit (Cedarlane#CLAS10-1121) IgG.

### Flow cytometry

To identify the expression of NOD1 intracellularly, detached cells were resuspended in PBS and treated with Fixation and Permeation Reagent kit (eBioscience#00-5521-00) for 1 h at 4 °C. The cells were then washed and incubated with primary anti-NOD1 antibody (1:100, anti-human or anti-mouse) for 30 min at 4 °C. Subsequent to that, the cells were washed and incubated with the corresponding secondary FITC-coupled antibody (1:100) for 30 min at 4 °C in the dark. They were then immediately acquired with BD FACScan flow cytometer and analyzed with BD CellQuest software. Background florescence was controlled with cells incubated with secondary FITC antibody alone.

### Immunohistochemistry

Human colon cancer TMA from USBioLab (Maryland, USA) was deparaffinized using xylene and rehydrated using graded ethanol (100%, 95%, 70%, 50%) and distilled water. Antigen retrieval was performed using pH 6 citrate buffer in pressurized heater for 16 min. After cooling, samples were permeated using 2% Triton X-100/TBS for 30 min. Endogenous peroxidase, avidin and biotin were blocked using 3% hydrogen peroxide, avidin and biotin blocking solutions in 2% normal horse serum (NHS) (Vector Labs #SP-2001, #S-2000). TMAs were incubated with anti-NOD1 (1:200) or NHS for vehicle control at 4 °C overnight. The next day, after washing with 0.05% TBST, biotinylated secondary antibody (Vector Labs #BA-1100), streptavidin-HRP, DAB chromogen (Vector Labs VECTASTAIN Elite ABC kit #PK-6100, ImmPACT DAB Peroxidase substrate #SK-4105) and Hematoxylin (Sigma-Aldrich) were sequentially added before slide dehydration in graded ethanol and xylene and coverslip with Permount mounting medium (Fischer Scientific). After drying, the specimens were examined twice by a Royal College-certified pathologist (Dr. R. McClure) who is blinded and have no prior knowledge of the study. Each core staining intensity was scored as “3-Strong-staining”, “2-Some-staining” or “1-No-staining”.

### Intravital fluorescent microscopy (IVM)

We previously described this technique (Hsu et al., 2011). In brief, wildtype cancer cells, treated with DMSO (control), C12 and ML130 + C12, were stained with 25 mmol/L CSFE and administed to 6–8 week-old C57/BL6 male mice (CharlesRiver) via intrasplenic injection. Fluorescently-labeled cancer cells were visualized at the liver edge and were counted in 10 random microscope fields. Observer was blinded to treatments. To ensure that the results are not due to a single cell line or species alone, human (HT29) and murine (MC38) colon cancer cells were both tested.

### *In vivo* hepatic metastasis assay

Seven week old wildtype C57/BL6 male mice (CharlesRiver) were treated with intraperitoneal injection of DMSO (control), C12-iE-DAP (100 μg/kg), ML130 (50 mmol/L/kg) for one hour. They were then anesthetized using isoflurane. Spleens were exposed through a small abdominal incision. 5 × 10^5^ of correspondingly treated MC38 cells, resuspended in 100 μL PBS, were injected intrasplenically and the mice were splenectomized two minutes later. Mice were sacrificed 21 days post-surgery. Total surface liver metastases were counted. Observer was blinded to the treatment groups.

### Extracellular matrix adhesion assay

We previously described this technique (Hsu et al., 2011). In brief, treated cancer cells were loaded onto 96-well plates coated collagen I, collagen IV, or fibronectin. After incubation and washing, the plate was stained with 1% crystal violet (CV), and then solubilized with methanol for absorbance scanning using BioTek ELx808 plate reader at a wavelength of 595 nm.

### NOD1 knockdown

To more accurately delineate the effect of NOD1, stable NOD1-knockdown sub-cultures of HT29 were created using SMARTvector 2.0 Lentiviral hEF1a-TurboRFP shRNA vector (ThermoScientific) per manufacture’s transfection protocol (https://dharmacon.horizondiscovery.com/uploadedFiles/ Resources/smartvector-constitutive-manual.pdf). A total of three constructs, including SH09-004398-01-10 (Sh1) TAACGTCTGGTTGACTTTC, SH09-004398-03-10 (Sh3) TCTTCGCTTAGCACCTTTA, and SH09-004398-04-10 (Sh4) TGGTTGAAGCTTTCGACCT, as well as the corresponding non-targeting hEF1a-TurboRFP control (NTC) were used. Transduced cancer cells were selected based on their RFP expression and puromycin-resistance.

To ensure results of knockdown were not secondary to off-target viral effects, siRNA knockdown (SantaCruz sc-37280) of HT29 cell line was also created using the manufacture’s transfection protocol (http://datasheets.scbt.com/protocols/ siRNA_protocol.pdf). The siRNA consisted of a pool of three different siRNAs with sequences of GAAGAGCUGACCAAAUACA, CAGAACACGUCUCUA GAAA, and CGUGUUUGAUGGAUUAGUA.

### Cell lysis and immunoblotting

Following stimulation, cells were washed twice with ice-cold PBS and then lysed in ice-cold buffer previously described by Berube et al. (2009). Each lysate was submitted to SDS-PAGE (10%) electrophoresis and subsequently transferred to nitrocellulose membranes (pore size 0.45 μm) and immunoblotted with specific primary antibodies and DyLight 680 and 800 secondary antibodies. The protein blots were then scanned immediately with LiCor Imaging System and analyzed using Odyssey imaging software.

### Statistical analysis

Continuous variables were analyzed using *t*-test or one-way ANOVA with Dunette’s adjustment for multiple comparisons. All statistical comparisons within each experiment were made against the control or baseline. All continuous data are reported as mean (M) ± standard error (SEM). Matched categorical variables were analyzed using Cochran-Mantel-Haenszel nonparametric test. Graphs were prepared using Prism® GraphPad version 5. Two-tailed alpha level of 5% was chosen. Kaplan-Meier plots and Cox regression analysis were prepared using JMP® version 11. TCGA NOD1 survival data was acquired and analyzed using OncoLnc® (http://www.oncolnc.org/).

## Electronic supplementary material

Below is the link to the electronic supplementary material.

Supplementary material 1 (PDF 403 kb)
