# Supplementary material for: Activation of the pattern recognition receptor NOD1 augments colon cancer metastasis
Source: Protein Cell. 2020 Jan 19;11(3):187–201. doi: 10.1007/s13238-019-00687-5 (PMC7026222; doi:10.1007/s13238-019-00687-5)
Supplement: Supplementary file 1 — Supplementary material 1 (PDF 403 kb) [file 13238_2019_687_MOESM1_ESM.pdf]

Supplementary Figures

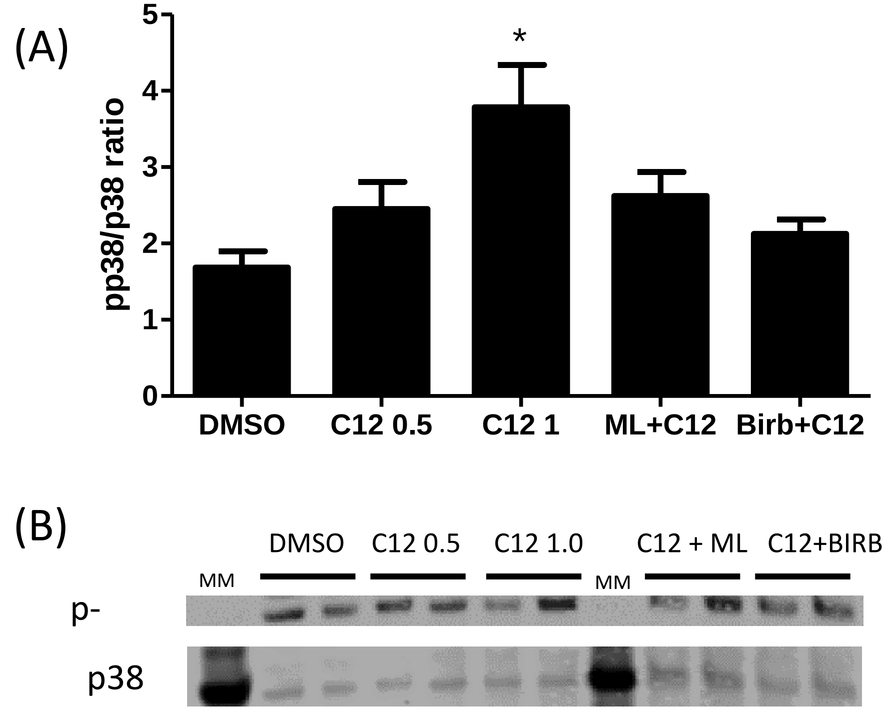

Supplementary Figure S1: NOD1 activation by C12-iE-DAP in murine MC38 colon cancer cell line promotes p38 MAPK phosphorylation. There is an increase in pp38/p38 ratio with increasing concentration of C12-iE-DAP compared to baseline. This increase is abrogated in the presence of ML130 and BIRB0796. "*" denotes significance (p = 0.05). M ±SEM is reported. All comparisons are made with respect to the baseline (BL) control. "MM" represents molecular marker.

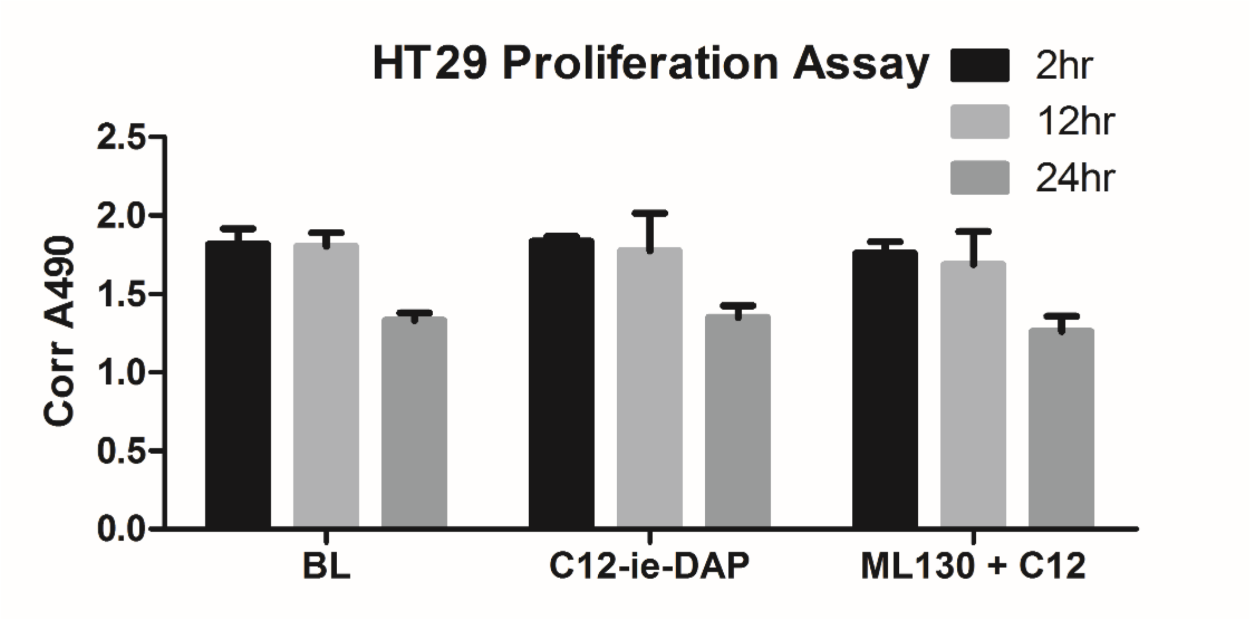

Supplementary Figure S2: Effects of NOD1 activation are independent of cancer cell proliferation. Level of MTS remains the same across baseline, C12-iE-DAP, and ML130+C12-iE-DAP at all time points tested ($p = 0.4$). M±SEM is reported. All comparisons are made with respect to the baseline (BL) control.

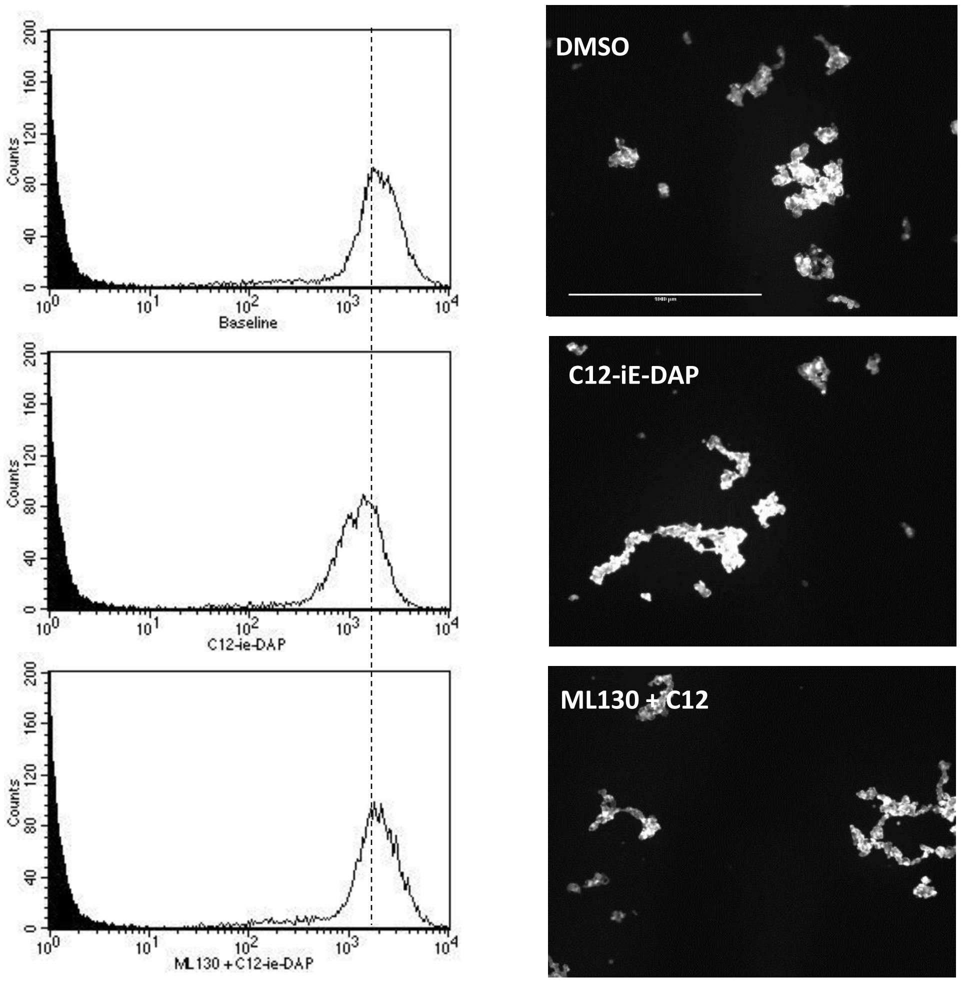

Supplementary Figure S3: NOD1 activation did not decrease HT29 apoptosis during the adhesion-free phase. Circulating tumor cells that can evade apoptosis and host detection can potentially seed distant organs compared to their apoptotic peers. The anoikis assay performed demonstrates no significant difference in apoptosis in HT29 cells upon NOD1 activation and inhibition (p = NS).
